# Supplementary material for: Diosgenin Loaded Polymeric Nanoparticles with Potential Anticancer Efficacy
Source: Biomolecules. 2020 Dec 16;10(12):1679. doi: 10.3390/biom10121679 (PMC7765552; doi:10.3390/biom10121679)
Supplement: Supplementary file 1 [file biomolecules-10-01679-s001.pdf]

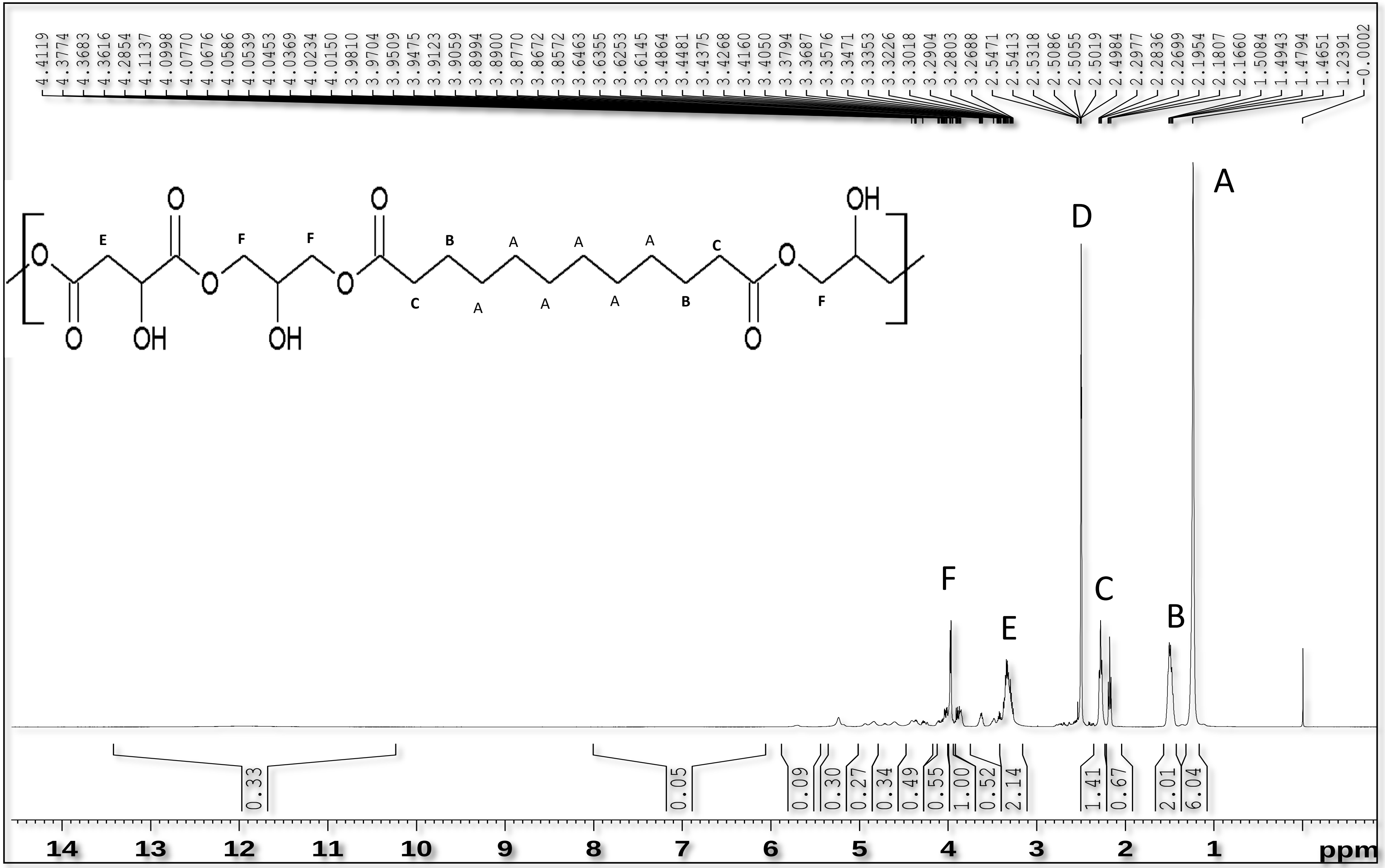

Figure-S1-The molecular structure of the polymer (PGMD) was investigated by ^{1}H NMR (Bruker Advance II JEOL 500 MHz spectrometer) with DMSO-d_6 as a solvent and TMS as internal standard. The chemical shifts were recorded and are identified as shown the figure.

The ^{1}H NMR spectra of PGMD (Poly-glycerol-malic acid- dodecanedioic acid) in DMSO- d_6 solvent at ambient temperature. The free polymer in the solution showed different peaks according to its structure. The peak at 1.239 ppm corresponds to the methylene protons (-CH_2-). Similarly, other peaks lying in the range 1.5 ppm, 2.1 ppm, 2.29 ppm, 3.3-4 ppm depicts the methylene protons of the polymer. From the spectra it is evident that the individual components of the polymer (malic acid, glycerol and DDA) were successfully incorporated in the PGMD polymer.

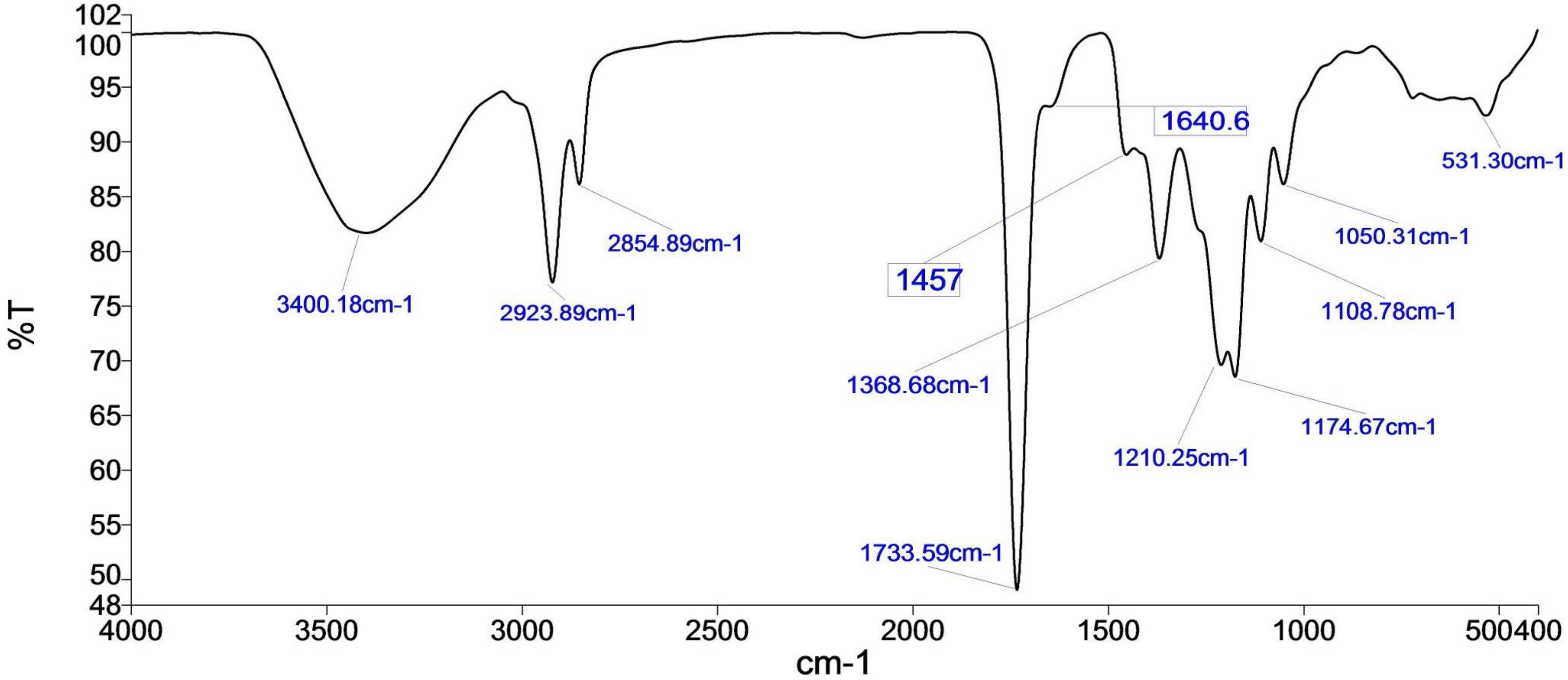

Figure S2-For the functional group analysis, vacuum dried samples of PGMD 7:3 was analysed by Fourier transform infrared spectroscopy (FTIR). The pellets was prepared along with KBr and scanned in the range of 4000 to 400 cm−1 against blank KBr pellet. The FT-IR studies of PGMD 7:3 polymer showed the presence of a C=O stretch at 17333.59 cm−1, which is a typical of ester bonds.

Size Distribution Report by Intensity

v2.2

Malvern

Sample Details

Sample Name: Dios np 6;4

SOP Name: mansettings.nano

General Notes:

**File Name:**	Nikita.dts	**Dispersant Name:**	Water
**Record Number:**	209	**Dispersant RI:**	1.330
**Material RI:**	1.59	**Viscosity (cP):**	0.8872
**Material Absorbtion:**	0.010	**Measurement Date and Time:**	22 May 2017 10:31:53 AM

System

**Temperature (°C):**	25.0	**Duration Used (s):**	60
**Count Rate (kcps):**	397.4	**Measurement Position (mm):**	4.65
**Cell Description:**	Disposable sizing cuvette	**Attenuator:**	7

Results

			Size (d.nm):	% Intensity:	St Dev (d.n...
**Z-Average (d.nm):**	121.4	**Peak 1:**	181.4	100.0	125.9
**PdI:**	0.270	**Peak 2:**	0.000	0.0	0.000
**Intercept:**	0.937	**Peak 3:**	0.000	0.0	0.000

Result quality : Good

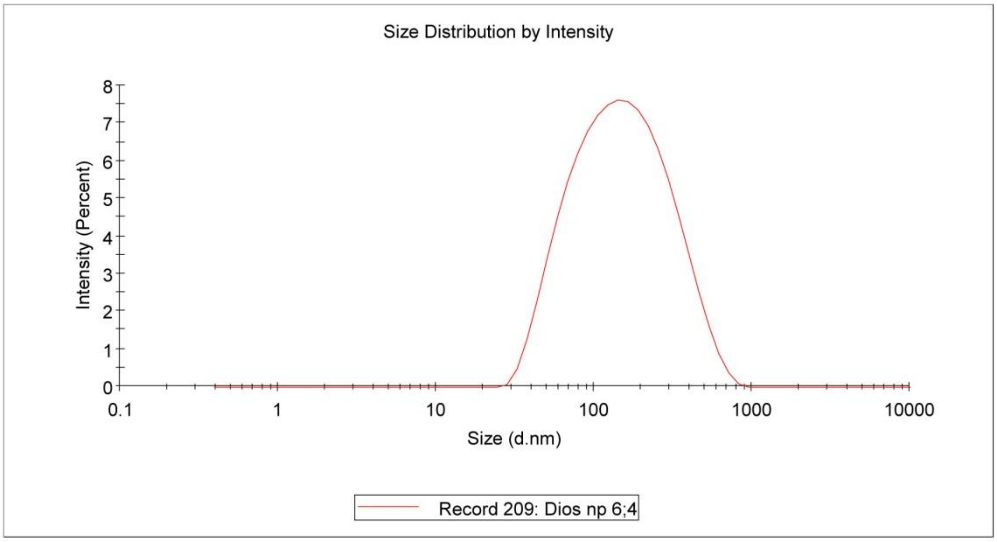

Figure S3: Represents the Size and PDI analysis of PGMD-DG 6:4 NP

Size Distribution Report by Intensity

v2.2

Malvern

Sample Details

Sample Name: dios nps 7-3

SOP Name: mansettings.nano

General Notes:

**File Name:**	Nikita.dts	**Dispersant Name:**	Water
**Record Number:**	210	**Dispersant RI:**	1.330
**Material RI:**	1.59	**Viscosity (cP):**	0.8872
**Material Absorbtion:**	0.010	**Measurement Date and Time:**	29 May 2017 11:08:40 AM

System

**Temperature (°C):**	25.0	**Duration Used (s):**	70
**Count Rate (kcps):**	216.1	**Measurement Position (mm):**	4.65
**Cell Description:**	Disposable sizing cuvette	**Attenuator:**	6

Results

			Size (d.nm):	% Intensity:	St Dev (d.n...
**Z-Average (d.nm):**	133.6	**Peak 1:**	147.3	100.0	53.27
**PdI:**	0.152	**Peak 2:**	0.000	0.0	0.000
**Intercept:**	0.933	**Peak 3:**	0.000	0.0	0.000

Result quality : Good

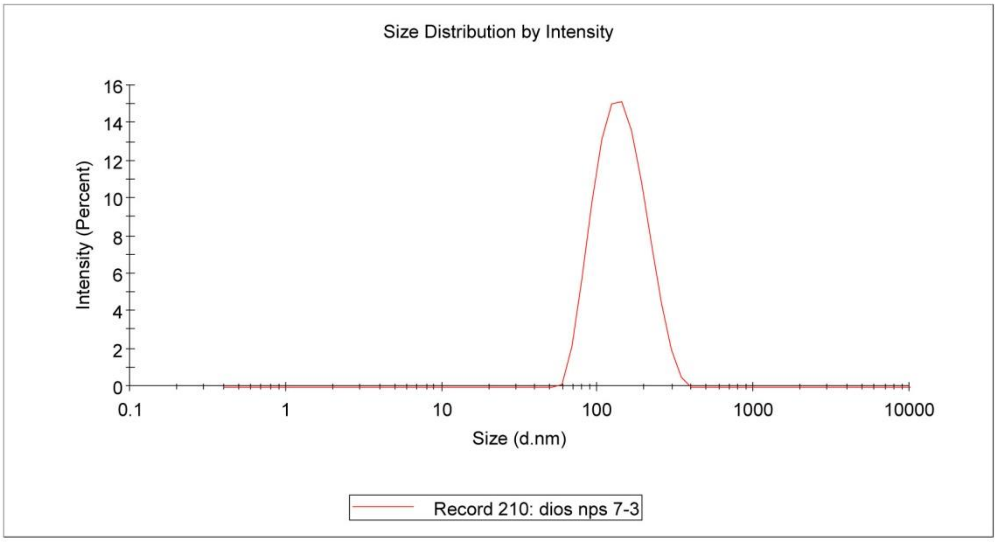

Figure S4: Represents the Size and PDI analysis of PGMD-DG 7:3 NP

Size Distribution Report by Intensity

v2.2

Malvern

Sample Details

**Sample Name:**	pgmd 6-4
**SOP Name:**	mansettings.nano
**General Notes:**	

**File Name:**	Nikita_Zeta (2).dts	**Dispersant Name:**	Water
**Record Number:**	274	**Dispersant RI:**	1.330
**Material RI:**	1.59	**Viscosity (cP):**	0.8872
**Material Absorbtion:**	0.010	**Measurement Date and Time:**	29 May 2017 12:06:15 PM

System

**Temperature (°C):**	25.0	**Duration Used (s):**	70
**Count Rate (kcps):**	211.3	**Measurement Position (mm):**	4.65
**Cell Description:**	Disposable sizing cuvette	**Attenuator:**	5

Results

			Size (d.nm):	% Intensity:	St Dev (d.n...
**Z-Average (d.nm):**	114.9	**Peak 1:**	135.2	100.0	56.67
**PdI:**	0.137	**Peak 2:**	0.000	0.0	0.000
**Intercept:**	0.933	**Peak 3:**	0.000	0.0	0.000
**Result quality :**	Good				

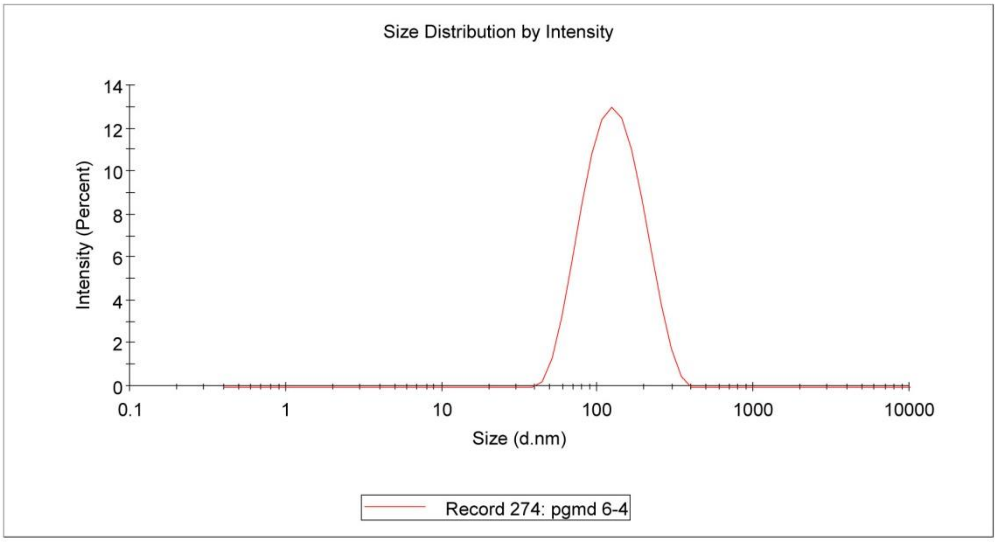

Figure S5: Represents the Size and PDI analysis of PGMD 6:4 NP

Size Distribution Report by Intensity

v2.2

Malvern

Sample Details

**Sample Name:**	Pgmd 7-3
**SOP Name:**	mansettings.nano
**General Notes:**	

**File Name:**	Nikita (1).dts	**Dispersant Name:**	Water
**Record Number:**	196	**Dispersant RI:**	1.330
**Material RI:**	1.59	**Viscosity (cP):**	0.8872
**Material Absorbtion:**	0.010	**Measurement Date and Time:**	29 May 2017 11:30:45 AM

System

**Temperature (°C):**	25.0	**Duration Used (s):**	70
**Count Rate (kcps):**	250.1	**Measurement Position (mm):**	4.65
**Cell Description:**	Disposable sizing cuvette	**Attenuator:**	5

Results

			Size (d.nm):	% Intensity:	St Dev (d.n...
**Z-Average (d.nm):**	111.6	**Peak 1:**	127.6	100.0	48.71
**PdI:**	0.111	**Peak 2:**	0.000	0.0	0.000
**Intercept:**	0.921	**Peak 3:**	0.000	0.0	0.000
**Result quality :**	Good				

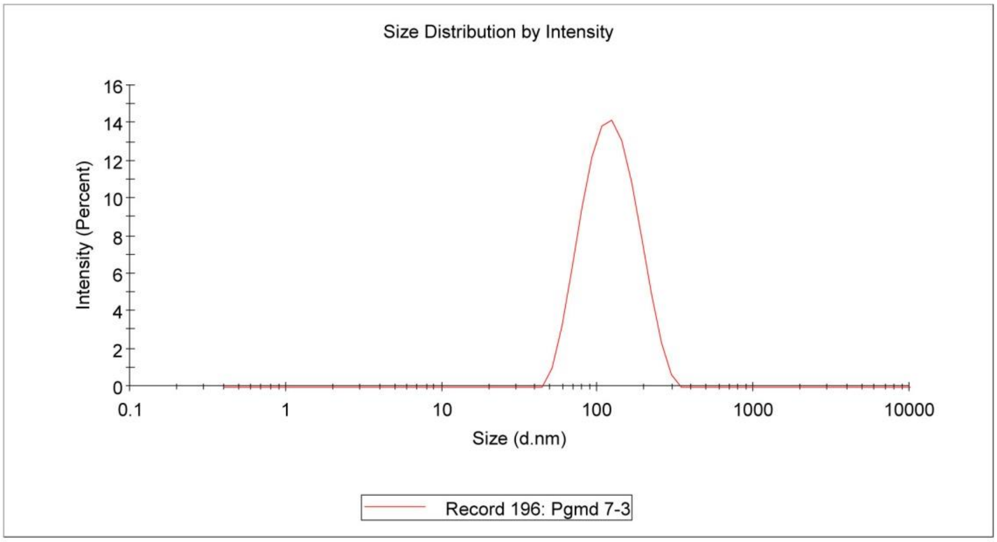

Figure S6: Represents the Size and PDI analysis of PGMD 7:3 NP
